# *PTENP1-AS* contributes to BRAF inhibitor resistance and is associated with adverse clinical outcome in stage III melanoma

**DOI:** 10.1038/s41598-021-89389-9

**Published:** 2021-05-26

**Authors:** Linda Vidarsdottir, Alireza Azimi, Ishani Das, Ingibjorg Sigvaldadottir, Aldwin Suryo Rahmanto, Andreas Petri, Sakari Kauppinen, Christian Ingvar, Göran Jönsson, Håkan Olsson, Marianne Frostvik Stolt, Rainer Tuominen, Olle Sangfelt, Katja Pokrovskaja Tamm, Johan Hansson, Dan Grandér, Suzanne Egyházi Brage, Per Johnsson

**Affiliations:** 1 Department of Oncology and Pathology, Karolinska Institutet, Stockholm, Sweden; 2 Department of Cell and Molecular Biology, Karolinska Institute, Stockholm, Sweden; 3 Center for RNA Medicine, Department of Clinical Medicine, Aalborg University, Copenhagen, Denmark; 4 Department of Clinical Sciences, Lund University, Lund, Sweden

**Keywords:** Cancer, Molecular biology

## Abstract

BRAF inhibitors (BRAFi) selectively target oncogenic BRAF^V600E/K^ and are effective in 80% of advanced cutaneous malignant melanoma cases carrying the V600 mutation. However, the development of drug resistance limits their clinical efficacy. Better characterization of the underlying molecular processes is needed to further improve treatments. We previously demonstrated that transcription of *PTEN* is negatively regulated by the *PTEN* pseudogene antisense RNA, *PTENP1-AS*, and here we investigated the impact of this transcript on clinical outcome and BRAFi resistance in melanoma. We observed that increased expression levels of *PTENP1-AS* in BRAFi resistant cells associated with enrichment of EZH2 and H3K27me3 at the *PTEN* promoter, consequently reducing the expression levels of *PTEN*. Further, we showed that targeting of the *PTENP1-AS* transcript sensitized resistant cells to BRAFi treatment and that high expression of *PTENP1-AS* in stage III melanoma correlated with poor survival. Collectively, the data presented here show that *PTENP1-AS* is a promising target for re-sensitizing cells to BRAFi and also a possible prognostic marker for clinical outcome in stage III melanoma.

## Introduction

Although cutaneous malignant melanoma is a molecularly diverse disease, approximately 50% carry activating mutations in the serine/threonine protein kinase BRAF. The majority of BRAF mutations are represented by a valine (V) to glutamic acid (E) or lysine (K) substitution at position 600 (BRAF^V600E/K^)^1^. These missense mutations occur in the BRAF kinase domain, which results in constitutively active BRAF and subsequently activated MAPK signaling. Drug development efforts using a targeted approach have successfully led to the development of small inhibitory molecules (BRAFi, i.e. vemurafenib, dabrafenib, encorafenib), which specifically target the oncogenic BRAF^V600E/K^. While 80% of patients with advanced melanoma carrying a BRAF^V600E/K^ mutation initially respond well to this treatment, resistance emerges within a median of 6–7 months of treatment^2^. Although the time to emergence of resistance can be prolonged to 11 months when combined with MEK inhibitors, the 5-year overall survival rate is only 34% for the combined treatment^3^. In recent years there has been a growing understanding of the underlying molecular mechanisms involved in acquired BRAFi resistance^4,5^ and activation of the PI3K/AKT pathway due to loss of PTEN has been found to contribute to this process^6^. Therefore, better molecular understanding of the mechanisms that contribute to suppression of PTEN is desired and might help to identify novel approaches that could circumvent or delay the onset of resistance.

Approximately 14,000 pseudogenes have been identified in the human genome and roughly 900 of these have been reported to be transcribed into non-coding RNAs (ncRNAs)^7^. Molecular functions have been characterized for only a handful of these pseudogenes^8–11^, for instance through microRNA (miRNA) sponging^10^ and *trans* acting antisense RNAs (asRNA)^8^. One of these, the *PTEN* pseudogene,  *PTENP1* (also known as *PTENpg1* and *PTENΨ*), is divergently transcribed into sense (*PTENP1-S*) and asRNA (*PTENP1-AS*) transcripts. While *PTENP1-S* promote expression of *PTEN* by functioning as a miRNA sponge for *PTEN* related miRNAs^10^, *PTENP1-AS* acts as a transcriptional suppressor by inducing epigenetic alterations at the *PTEN* promoter^8,12^. Interestingly, loss of PTEN expression has been found during the development of resistance to BRAFi^6^ and melanoma metastasis^13,14^, but the involvement of *PTENP1-AS* remains unknown.

In this study, our aim was to characterize the impact of *PTENP1-AS* on clinical outcome in stage III melanoma and BRAFi treatment efficacy. We observe that the expression of *PTENP1-AS* is induced in BRAFi resistant sublines in relation to the parental sensitive cell line. This coincides with transcriptional suppression of *PTEN,* likely through the recruitment of EZH2 and subsequent formation of H3K27me3 at the *PTEN* promoter. Finally, we also demonstrate that expression of *PTENP1-AS* is increased in tumor samples from stage III melanoma patients with poor survival. In summary, our study provides insights into the involvement of *PTENP1-AS* in resistance to BRAFi treatment.

## Results

### Inverse correlation of *PTEN* and *PTENP1-AS* in BRAFi resistant A375 sublines

**Figure 1:**
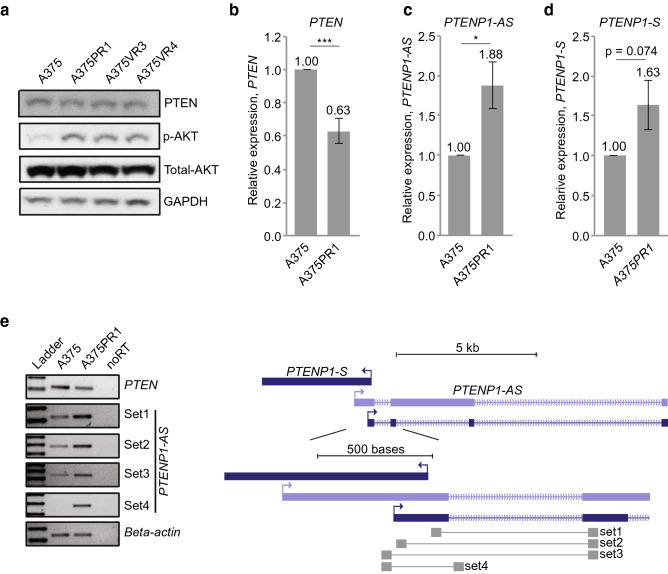
Expression levels of PTEN and *PTENP1*-lncRNAs in BRAFi sensitive and resistant cell lines. (**a**) Western blot analysis presenting the expression of PTEN, total AKT and p-AKT in A375 and the BRAFi resistant sublines. (**b**–**d**) qRTPCR analysis of (**b**) *PTEN,* (**c**) *PTENP1-AS* and (**d**) *PTENP1-S* in A375 and the BRAFi resistant A375PR1 subline (n = 3, p-values represent a two-tailed student’s t-test). (**e**) Semi-qRTPCR assay showing different isoforms of the *PTENP1-AS* transcripts with a scheme showing primer binding sites.

Previous studies suggested downregulation of *PTEN* to be important upon the development of resistance to BRAFi^6,13^. Based on these reports, we set out to investigate if *PTENP1-AS* was involved in this process through transcriptional suppression of *PTEN*. To do this, a series of BRAFi resistant melanoma sublines were obtained by culturing the BRAFi sensitive A375 cell line in increasing doses of BRAFi (A375PR1 (resistant to PLX4720), A375VR3 and A375VR4 (both resistant to vemurafenib))^15^. Compared to the parental A375 cells, IC_50_ measurements for the A375PR1 and A375VR4 cell lines confirmed roughly six- to eight-fold increased tolerance to vemurafenib (Supplementary Fig. S1), thus in agreement with a previous study^15^. We next measured PTEN expression using Western blot and qRTPCR and observed downregulation of PTEN at the protein (Fig. 1a, Supplementary Fig. S2a) as well as mRNA (Fig. 1b, Supplementary Fig. S2b) levels. Importantly, the negatively regulated downstream target of PTEN, p-AKT, showed a corresponding increase in phosphorylation compared to the parental A375 cell line (Fig. 1a, Supplementary Fig. S2a).

We next measured levels of *PTENP1*-encoded transcripts and identified a significant upregulation of *PTENP1-AS* in all three resistant sublines (Fig. 1c, Supplementary Fig. S2c) and also a modest, although non-significant, induction of *PTENP1-S* (Fig. 1d, Supplementary Fig. S2d). Since previous studies had identified multiple isoforms of *PTENP1-AS* with distinct regulatory functions^8^, we carefully investigated different isoforms in one of the sublines (A375PR1). Multiple primer sets for the different variants of *PTENP1-AS* were assessed on semi-qRTPCR and revealed increased expression of all isoforms (Fig. 1e, Supplementary Fig. S3). Notably, unspliced RNA levels of *PTENP1-AS* was also found to be induced, indicating that *PTENP1-AS* is activated at the transcriptional level. Collectively, these initial observations motivated us to further elucidate the role of *PTENP1-AS* in resistance to BRAFi.

### *PTENP1-AS *suppresses *PTEN* in BRAFi resistant cells through chromatin remodeling

**Figure 2:**
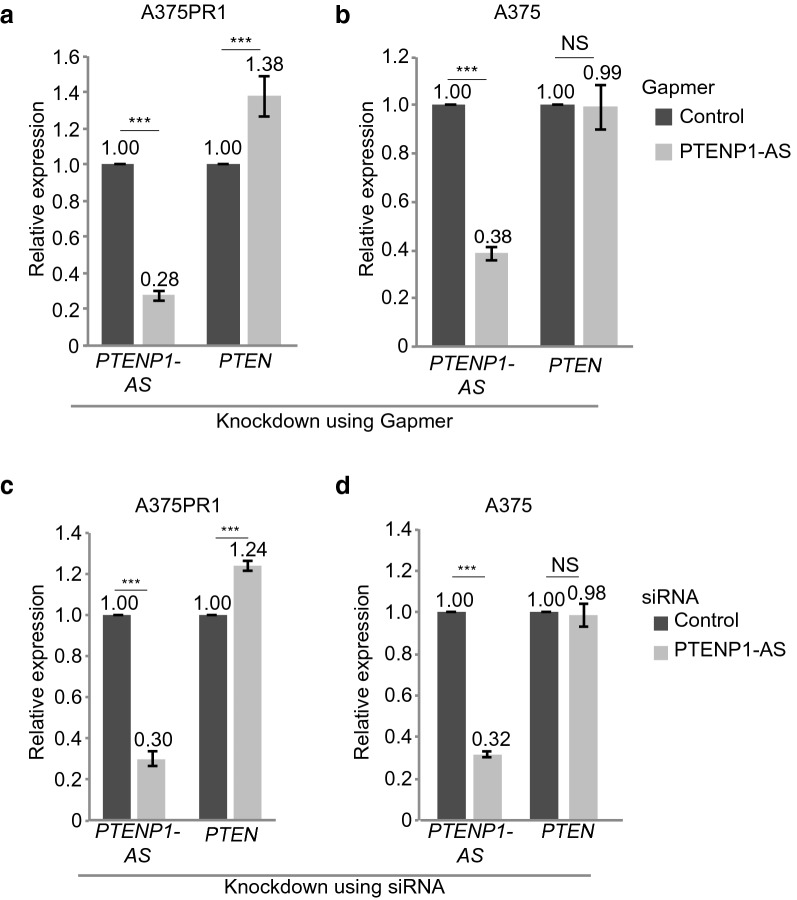
Effect on *PTEN* expression upon knockdown of *PTENP1-AS*. (**a**,**b**) qRTPCR measuring the expression levels of *PTENP1-AS* and *PTEN* in (**a**) A375PR1 or (**b**) A375 cells upon knockdown of *PTENP1-AS* using gapmer ASO. (**c**,**d**) qRTPCR measuring the expression levels of *PTENP1-AS* and *PTEN* in (**c**) A375PR1 or (**d**) A375 cells upon knockdown of *PTENP1-AS* using siRNA. (**a**–**d**) n = 3, p-values represent two-tailed student’s t-tests.

To investigate the molecular interplay between *PTENP1-AS* and *PTEN* in greater detail, we focused, from here, on the A375PR1 subline. We designed a gapmer antisense oligonucleotide (ASO) (Fig. 2a,b) and also an siRNA targeting the *PTENP1-AS* transcript (Fig. 2c,d) and confirmed knockdown in A375 as well as in A375PR1 cells using qRTPCR. Intriguingly, knockdown of *PTENP1-AS* only induced the expression of *PTEN* in the A375PR1 cells, while *PTEN* remained unaffected in the vemurafenib sensitive A375 cells (Fig. 2a–d), suggesting that *PTENP1-AS* is predominantly active in the resistant subline. In contrast, no effect on *PTENP1-S* expression levels was observed in either cell line upon knockdown of *PTENP1-AS* (Supplementary Fig. S4).

**Figure 3:**
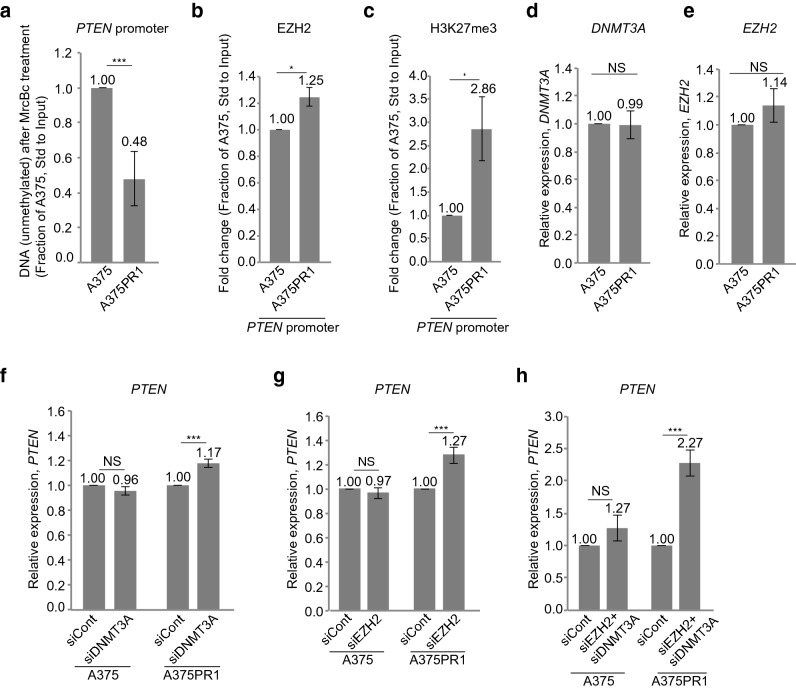
Characterization of the *PTEN* promoter in A375 and A375PR1 cell lines. (**a**) qRTPCR measuring *PTEN* promoter CpG methylation, as determined by the remaining (unmethylated) DNA upon treatment with the methyl-cytosine-specific restriction enzyme McrBc (n = 3). (**b–c**) ChIP followed by qRTPCR analysis measuring the levels of (**b**) EZH2 and (**c**) H3K27me3 at the *PTEN* promoter in A375 and A375PR1 cell lines (n = 3). (**d**–**e**) qRTPCR measuring baseline expression levels of (**d**) *DNMT3A* and (**e**) *EZH2* in A375 and A375PR1 cell lines (n = 3). (**f**–**g**) qRTPCR measuring the expression levels of *PTEN* following siRNA induced knockdown of (**f**) *DNMT3A* and (**g**)* EZH2* in A375 and A375PR1 cell lines (n > 3). (**h**) qRTPCR measuring the expression levels of *PTEN* following simultaneous siRNA-induced knockdown of *EZH2 and DNMT3A* in A375 and A375PR1 cell lines (n = 3). (**a**–**h**; p-values represent a two-tailed student’s t-test).

We next asked if suppression of *PTEN* in the A375R1 subline was due to epigenetic changes at the *PTEN* promoter since high promoter methylation has been commonly observed in melanoma and is associated with poor clinical outcome^16^. *PTEN* promoter CpG methylation was assessed by the methyl-cytosine-specific McrBc restriction enzyme. qRTPCR showed increased enzymatic cleavage in the A375PR1 subline, compared to the parental A375 cells (Fig. 3a), thus suggesting increased *PTEN* promoter CpG methylation in the resistant subline. Since we have previously reported *PTENP1-AS* to modulate transcription of *PTEN* through the recruitment of EZH2 and DNMT3A^8,12^, we next measured levels of EZH2 and H3K27me3 at the *PTEN* promoter. Chromatin immunoprecipitation (ChIP) followed by qRTPCR revealed increased levels of EZH2 and H3K27me3 at the *PTEN* promoter in the A375PR1 subline (Fig. 3b,c), while the baseline expression levels of *DNMT3A* and *EZH2* were similar in the resistant and parental cell lines as measured by qRTPCR (Fig. 3d,e). To further interrogate their role in suppression of *PTEN*, *EZH2* and *DNMT3A* were depleted using siRNAs. Although individual siRNA-mediated knockdown only resulted in a modest induction of *PTEN* (Fig. 3f,g, Supplementary Fig. S5a,b), simultaneous knockdown generated over a twofold induction of *PTEN* in the A375PR1 cells, in contrast to the parental A375 cells, where the expression of *PTEN* was largely unchanged (Fig. 3h, Supplementary Fig. S5c,d). Altogether, these data suggest that suppression of *PTEN* is mediated by *PTENP1-AS* likely through the recruitment of EZH2 by a mechanism that may be exclusively active in the vemurafenib resistant cells.

### C/EBPB is a transcriptional regulator of *PTENP1-AS*

**Figure 4:**
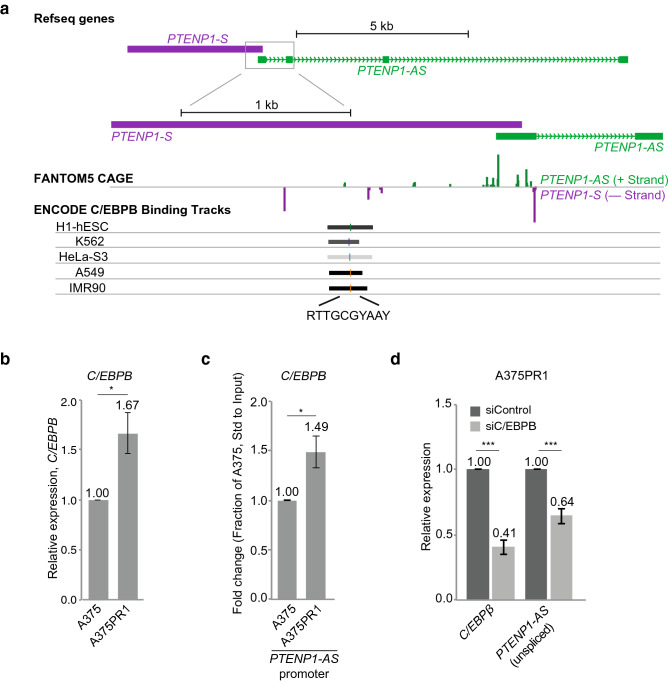
Transcriptional regulation of *PTENP1-AS* by C/EBPB. (**a**) A scheme showing binding of C/EBPB at the *PTENP1* locus (data retrieved from the Zenbu FANTOM5 CAGE and the UCSC genome browser) and the predicted DNA binding site for C/EBPB (R is an A/G, Y is C/T). (**b**) qRTPCR measuring the expression levels of *C/EBPB* (n > 3). (**c**) ChIP followed by qRTPCR analysis measuring the levels of C/EBPB at the *PTENP1-AS* promoter (n = 3). (**d**) qRTPCR measuring expression levels of *C/EBPB* and unspliced *PTENP1-AS* in A375PR1 cells upon siRNA-induced knockdown of *C/EBPB* (n = 3).

To explore mechanisms underlying transcriptional induction of *PTENP1-AS* (Fig. 1c), we took advantage of FANTOM5 CAGE data and ChIP-sequencing data from the UCSC Genome Browser and identified a binding site for the transcription factor (TF) C/EBPB close to the *PTENP1-AS* transcriptional start site (TSS) (Fig. 4a). Comparing A375PR1 to A375 cells, revealed an increased RNA expression of *C/EBPB* by qRTPCR (Fig. 4b), and ChIP further identified increased levels of C/EBPB at the *PTENP1-AS* promoter (Fig. 4c) in A375PR1 cells. Targeting *C/EBPB* with dsiRNA reduced the expression of unspliced *PTENP1-AS* in the A375PR1 cells (Fig. 4d), while no significant change was identified for *PTENP1-S* (Supplementary Fig. S6), thus suggesting that C/EBPB functions as a transcriptional activator of *PTENP1-AS*.

### *PTENP1-AS* is a clinically relevant target and a prognostic marker in melanoma

**Figure 5:**
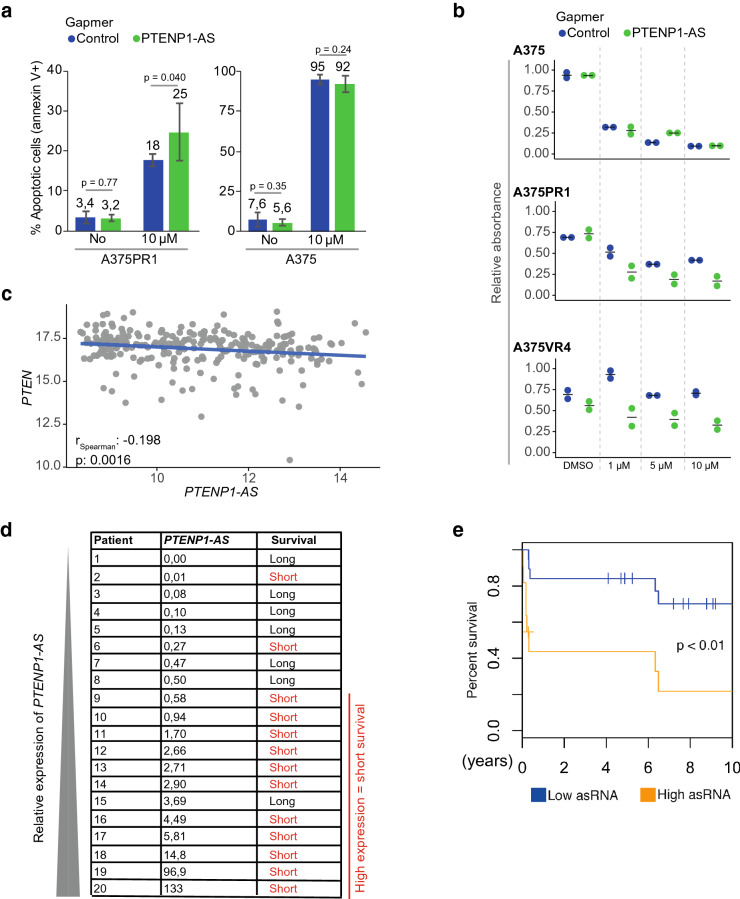
Manipulation of *PTENP1-AS* in BRAFi resistant sublines and evaluation of *PTENP1-AS* in metastatic melanoma samples. (**a**) Cell-death analysis by annexin V and propidium iodide staining upon knockdown of *PTENP1-AS* and vemurafenib treatment (n = 6). (**b**) Colony formation assays for A375, A375PR1 and A375VR4 cell lines measuring relative absorbance of crystal violet solution stained colonies upon gapmer ASO-induced knockdown of *PTENP1-AS* co-treated with various concentrations of vemurafenib. (**c**) Expression data (TCGA) for *PTEN* and *PTENP1-AS* from melanoma samples. (**d**) qRTPCR measuring expression levels of *PTENP1-AS* in a set of 20 first regional lymph node metastases from stage III melanoma patients. Based on clinical follow up data, the patients were categorized as long- or short-term survivors, > 60 months or ≤ 13 months, respectively. (**e**) Kaplan–Meier plot showing overall survival for patients with high (n = 12) or low (n = 17) expression levels of *PTENP1-AS*. Expression levels of *PTENP1-AS* was measured in first regional lymph node metastases from an independent set of 29 stage III melanoma patients using qRTPCR. The p-value represents a log-rank test.

We next explored if the resistant A375PR1 cells could be sensitized to BRAFi by targeting the *PTENP1-AS* transcript. Gapmer ASO-mediated suppression did not have an effect on the basal levels of cell death but sensitized resistant cells to treatment with 10 μM of vemurafenib (Fig. 5a), as measured by annexin V and propidium iodide staining. To further validate this effect, we performed a colony formation assay and also included a second resistant subline, A375VR4. While gapmer ASO-mediated knockdown of *PTENP1-AS* had minor effect on colony formation in parental A375, both resistant sublines were clearly sensitized to vemurafenib, in particular at lower concentrations (1 μM) (Fig. 5b, Supplementary Fig. S7).

We finally set out to investigate the relevance of *PTENP1-AS* in tumors from melanoma patients. The expression levels of *PTEN* and *PTENP1-AS* were first assessed in melanoma samples from the TCGA (The Cancer Genome Atlas program) where although weak but a significant negative correlation was observed (r = − 0.20, p = 1.6e^−3^, Fig. 5c). The expression of *PTENP1-AS* was next determined in lymph node metastases from a cohort of 20 stage III melanoma patients. The samples were initially chosen to include two groups of patients, with either long (≥ 60 months) or short (≤ 13 months) overall survival^17^, and the expression of *PTENP1-AS* was determined by qRTPCR. Notably, 9/10 (90%) patients having tumors with high expression of *PTENP1-AS* (median cut-off) had poor survival, while low expression of *PTENP1-AS* seemed to be indicative of prolonged survival, although not reaching significance (p = 0.06) (Fig. 5d). This motivated us to evaluate the expression of *PTENP1-AS* in an independent second cohort of stage III melanoma metastasis samples. Lymph node metastases from 29 stage III melanoma patients were analyzed for the expression of *PTENP1-AS*. The patient samples from the second cohort were divided in high (n = 12) or low (n = 17) expression of *PTENP1-AS*, as determined by qRTPCR, and a survival plot was generated (Fig. 5e). A significant difference in overall survival was observed between patients with high and low expression of *PTENP1-AS*. In summary, the expression of *PTENP1-AS* appears to be a promising prognostic marker for clinical outcome, where increased expression of *PTENP1-AS* correlates with poor overall survival.

## Discussion

The data presented in this study suggest a role for *PTENP1-AS* in the development of resistance to BRAFi. On the basis of our data, we suggest that expression of *PTENP1-AS* is induced by the TF C/EBPB in BRAFi resistant melanoma cell lines, which results in transcriptional suppression of *PTEN* through the recruitment of EZH2 with subsequent formation of H3K27me3 (Fig. 3b,c) and DNA methylation of the *PTEN* promoter (Fig. 3a). Moreover, we find that expression of *PTENP1-AS* predicts clinical outcome in stage III melanoma patients, where high expression of *PTENP1-AS* in first regional lymph node metastases correlates with poor overall survival. In summary, our findings bring important information about molecular functions of *PTENP1-AS* upon development of BRAFi resistance and better understanding of this pathway could reveal novel approaches to re-sensitize drug resistant cells.

Our data are consistent with our previous findings demonstrating that *PTENP1-AS* is a negative regulator of *PTEN* through chromatin remodeling of the *PTEN* promoter^8^. Targeting the *PTENP1-AS* transcript using gapmer ASOs and siRNAs generate a moderate induction of *PTEN* exclusively in the resistant subline (Fig. 2), thus demonstrating that the *PTENP1-AS* transcript does not act as an on/off switch for *PTEN* expression, but rather causes subtle variations of transcription in a phenotype-dependent manner^8^. These variations of *PTEN* expression are within physiological relevant levels where a modest decrease of 20% have been shown to increase cancer susceptibility^18^.

Our data is also consistent with previous RNA-sequencing studies showing induction of *C/EBPB* in BRAFi resistant cells^19^. However, these previous studies did not report *PTEN* or *PTENP1-AS* as candidate genes involved in BRAFi resistance, possibly due to the applied thresholds since the cut-off was set to a fold change ≥ 2. Based on our study, both *PTEN* and *PTEN1-AS* are likely to be excluded from such an analysis when these thresholds are applied.

Several *PTENP1*-encoded lncRNAs with opposing functions have been described. While the miRNA sponge model suggests concordant expression of *PTEN* and *PTENP1-S*^10^, we have not observed this phenomenon in the present study, despite a modest induction of *PTENP1-S* (Fig. 1d, Supplementary Fig. S2d). Instead, the *PTENP1-AS* transcript appears to be the dominant regulator of *PTEN* expression under these conditions. However, this does not exclude that miRNA sponging could take place as well. A regulatory mechanism, where translation as well as transcription of *PTEN* is regulated through lncRNAs encoded by the *PTENP1* locus is still a plausible scenario. Subtle variations of *PTEN* expression have previously been reported to be associated with cancer susceptibility^18^, suggesting that strict and highly ordered regulation of *PTEN* expression is crucial for evading carcinogenesis. It is therefore likely that *PTEN* is regulated at several different levels. Deletions of *PTENP1* have been reported in melanoma cell lines and tissues, supporting that suppression of *PTEN* also occurs at the post-transcriptional level through miRNA-mediated regulation^20^. It is also likely that tissue and cell type specific expression levels of i.e. DNMT3A, EZH2 and miRNAs dictate the regulatory outcome of *PTENP1*-encoded lncRNAs, and *PTENP1-S* may also be involved in sponging of miRNAs related to other mRNAs beyond *PTEN*^21^.

Notably, this study also shows that the suppression of *PTEN* in BRAFi resistant cells may be reversible through targeting of *EZH2, DNMT3A* as well as *PTENP1-AS*. The individual knockdown of *EZH2* or *DNMT3A* did not re-activate the expression of *PTEN* to the same extent as a simultaneous knockdown of these proteins (Fig. 3f–h). We speculate that this is due to incomplete knockdown of *DNMT3A* and *EZH2* where some active protein complexes may still be present under these circumstances (Supplementary Fig. S5). An interaction between EZH2 and DNMT3A have been demonstrated^22^ and the combined knockdown of *EZH2* and *DNMT3A* may in this case be more efficient in reducing these protein complexes. Importantly, the induction of *PTEN* is more prominent in the A375PR1 cells than in the parental cells, indicative of a therapeutic window for re-activation of *PTEN* in BRAFi resistant melanoma cells. In support of this, we show that manipulation of the *PTENP1-AS* pathway re-sensitized the A375PR1 and A375VR4 resistant cells to treatment with vemurafenib (Fig. 5a,b and Supplementary Fig. S7).

We evaluated the expression of *PTENP1-AS* in regional lymph node metastases of stage III melanoma and found that high expression correlated with poor survival (Fig. 5d,e). This indicates that *PTENP1-AS* may be involved in melanoma tumor progression and is not only important during the development of resistance to BRAFi. Also, development of a particular type of resistance to vemurafenib may depend on the pre-existing factors/pathways in the tumors. High expression of *PTENP1-AS* could, for example, indicate that the tumor is less likely to respond to treatment and loss of *PTEN* expression has previously been linked to metastasis^13^. Therefore, one may speculate that *PTENP1-AS* is involved in such inactivation and consequently enhance initiation of distant metastasis. Additional studies will be required to better understand the functional role and importance of *PTENP1-AS* in melanoma progression and drug resistance i.e. by taking patient samples pre- and post- treatment with BRAFi.

In summary, we have identified *PTENP1-AS* to be involved both in melanoma tumor progression and in development of resistance to vemurafenib. The data presented here show that *PTENP1-AS* is not only a promising target for re-activation of *PTEN*, but also a possible prognostic marker for clinical outcome in stage III melanoma.

## Materials and methods

### Cell cultures

A375 cells were purchased from ATCC (CRL-1619). A375, A375PR1, A375VR3 and A375VR4 cell lines were cultured in 5% CO_2_ at 37 °C in MEM supplemented with 10% heat-inactivated FBS, 2 mM glutamine, 0.1 mM non-essential amino acids, 1 mM sodium pyruvate, 50 μg/ml of streptomycin and 50 μg/ml of penicillin. As previously described^15^, resistant A375 cell lines had been generated by repeated exposures to increasing concentrations of vemurafenib (A375VR3 and A375VR4) or its analog PLX4720 (A375PR1).

### RNA extraction and cDNA

RNA was extracted using the RNA NucleoSpin II kit (Macherey–Nagel) and treated with DNase (Ambion Turbo DNA-free, Life Technologies). DNase treated RNA (~ 500 ng) was used for the generation of cDNAs using M-MLV (Life Technologies) and a mixture of oligo(dT)_15_ with nanomers.

### Semi-qRTPCR

PCR was performed by using the KAPA2G FAST mix (Kapa Biosystems) according to the manufacturer’s recommendations and by using the corresponding oligos in Supplementary Table 1.

### qRTPCR

qRTPCR was performed by using the KAPA 2G SYBR Green (Kapa Biosystems) on the Applied Biosystems 7900HT or the BioRad CFX96 Touch platform with the following cycling conditions: 95 °C for 3 min, 95 °C for 3 s, 60 °C for 30 s. The corresponding oligos for each gene is specified in Supplementary Table 1.

### siRNAs and gapmers

siRNAs and gapmer antisense oligonucleotides, ASOs, were ordered from the respective manufacturers (Supplementary Table 1) and transfected using lipofectamine 2000 (Life Technologies) according to the manufacturer’s recommendations. A final concentration of 10–40 nM was used for the siRNAs and gapmer ASOs and PLUS Reagent (Life Technologies), which was added for transfections of gapmers ASOs.

### Protein analysis

Samples were lysed in 50 mM Tris–HCl, pH 7.4, 1% NP-40, 150 mM NaCl, 1 mM EDTA, 1% glycerol, 100 μM vanadate, protease inhibitor cocktail and PhosSTOP (Roche Diagnostics GmbH). Lysates were subjected to SDS-PAGE using 4–12% acrylamide gels (Life Technologies) and transferred to PVDF membranes using the iBlot system (Life Technologies). The proteins were detected by western blot analysis by using an enhanced chemiluminescence system (Western Lightning–ECL, PerkinElmer). Antibodies used were specific for PTEN (Cell Signaling, cat. no. 9552, 1:1000), AKT (Cell Signaling, cat. no. 9272, 1:1000), phospho-AKT (Cell signaling, cat. no. 4060S, 1:1000) and β-actin (Sigma-Aldrich, cat. no. A5441, 1:5000). Membranes were first incubated for p-AKT followed by reprobing the same membranes for total AKT and PTEN.

### ChIP of EZH2, H3K27me3 and C/EBPB

ChIP assays were performed as previously described^8^. Briefly, the ChIP assay Kit (Upstate/Millipore) was used by crosslinking the cells in 1% formaldehyde for 10 min, quenched in 0.125 M Glycine for 5 min and lysed according to the manufacturer's recommendations. The samples/nuclei were sonicated with a Bioruptor Sonicator (Diagenode) at 30 s ON, 30 s OFF (setting = high) for a total of 18 cycles. The water was replaced with ice-cold water after every sixth cycle. The samples were diluted 1:10 in ChIP dilution buffer, pre-cleared and incubated overnight with the appropriate antibody. Salmon sperm DNA/Protein A-agarose (Upstate/Millipore) was used to pull down the antibody. The DNA was eluted in elution buffer (1% SDS, 100 mM NaHCO_3_), followed by reverse cross-linking at 65 °C overnight. The samples were RNase-A and protease-K treated and finally eluted using the Qiagen PCR purification kit (Qiagen). The following antibodies were used (4 μg/sample): H3K27me3 (Upstate/Millipore, cat. no. 17-622), EZH2 (Upstate/Millipore, cat. no. 07-689) and C/EBPB (Santa Cruz Biotechnology cat. no. sc-150).

### Assessment of methylated DNA

The methyl-cytosine-specific McrBc restriction enzyme McrBc (New England Biolabs) was used to cleave methylated DNA. Briefly, 200 ng DNA was digested with McrBc at 37 °C overnight and heat inactivated the next day at 65 °C for 1 h. Samples were run on a qPCR machine and standardized to uncut input. Delta CT values were converted to fold-change values and the ratio between A375PR1/A375 was calculated.

### PI-annexin V staining

The cells were harvested, washed twice in PBS and resuspended in 100 μL annexin V incubation buffer (10 mM HEPES/NaOH, pH 7.4, 140 mM NaCl, 5 mM CaCl_2_) containing 1% annexin V FLOUS (Roche Molecular Biochemicals) and 500 μg/μL PI stain. The samples were incubated for 15 min at room temperature followed by adding 400 μL of ice-cold annexin V incubation buffer and subsequently analyzed on a cytometry machine.

### IC50 measurements

4,000 cells per well were plated in 96 well plate format. The following day, cells were treated with the BRAFi vemurafenib (Selleckchem) at the indicated doses and further incubated for 72 h. The cell viability was then analyzed by MTS assay (Promega, Madison, WI, USA) according to manufacturer’s protocol followed by absorbance read at 490 nM using Tecan Spark 10 M plate reader (Tecan Trading AG, Switzerland). IC50 was analyzed using Graphpad Prism 8.0.

### Colony formation assay

1000 cells per well were plated in 6 well plates and transfected 24 h later by using lipofectamine RNAiMax (Life Technologies), at a final concentration of 20 nM of gapmer ASO (Control or PTENP1-AS). Vemurafib treatment (or DMSO control) was initiated the day after transfection, cells were let grown for another 8–10 days before being fixed in 4% buffered formaldehyde and stained with 0.05% crystal violet. To estimate number of colonies, crystal violet staining was dissolved in 100% methanol, diluted to 1:10 in PBS and absorbance was measured at 540 nM using a Tecan Spark 10 M plate reader.

### TCGA analysis

Gene expression profiles from GDC-TCGA (Genomic Data Commons—Cancer Genome Atlas) were utilized to correlate the expression of *PTENP1-AS* and *PTEN* in skin cutaneous melanoma (SKCM). We used the Xena browser (https://xenabrowser.net) to extract the RNA expression data for each gene, excluded samples with no expression of *PTENP1-AS* and *PTEN*, and correlated their expression using Spearman correlation (n = 251).

### Patient samples

First regional lymph node metastases from stage III melanoma patients that had not received any systemic treatment were collected. The RNA was extracted using the Qiagen RNeasy mini kit (Qiagen). The RNA was DNase treated on column according to the manufacturer’s recommendations. The expression of *PTENP1-AS* was analyzed using qRTPCR and standardized to *Beta-actin* (dCt) by using the corresponding oligos in Supplementary Table 1. ddCt was calculated by using the mean Ct value of all samples. High expression of *PTENP1-AS* (Fig. 5e) was defined as Ct < 30.

### Statistics

Two tailed Student’s T-test was used to determine statistical significance. Error bars represent the standard error of the mean. Statistical significance of the melanoma patient samples in Fig. 5e is evaluated using log-rank test. Throughout the paper, *P < 0.05; **P < 0.01; ***P < 0.005.

### Ethical approval and consent to participate

This study has been approved by the Research Ethics Committee of Karolinska Institutet (Dnr: 2006/1373-31/3) and the Regional Ethics Committee in Lund (Dnr 2013/101). All involved individuals have provided informed consent to be included in the study. All the study procedures were carried out in accordance with relevant guidelines.

## Supplementary Information

Supplementary Information.
