# Supplementary material for: PTENP1-AS contributes to BRAF inhibitor resistance and is associated with adverse clinical outcome in stage III melanoma
Source: Sci Rep. 2021 May 26;11:11023. doi: 10.1038/s41598-021-89389-9 (PMC8155038; doi:10.1038/s41598-021-89389-9)
Supplement: Supplementary file 1 — Supplementary Information. [file 41598_2021_89389_MOESM1_ESM.docx]

**SUPPLEMENTARY INFORMATION**

*PTENP1-AS* contributes to BRAF inhibitor resistance and is associated with adverse clinical outcome in stage III melanoma

Linda Vidarsdottir^1^, Alireza Azimi^1^, Ishani Das^1^, Ingibjorg Sigvaldadottir^1^, Aldwin Suryo Rahmanto^2^, Andreas Petri^3^, Sakari Kauppinen**^3^,** Christian Ingvar^4^, Göran Jönsson^4^, Håkan Olsson^4^, Marianne Frostvik Stolt^1^, Rainer Tuominen^1^, Olle Sangfelt^2^, Katja Pokrovskaja Tamm^1^, Johan Hansson^1^, Dan Grandér^1^, Suzanne Egyházi Brage^1,*^, Per Johnsson^2,*^

^*^ Corresponding authors: per.a.johnsson@ki.se

suzanne.egyhazi.brage@ki.se

**SUPPLEMENTARY FIGURES**

**Supplementary Figure 1.** *Dose response curves for vemurafenib in sensitive A375 cell line and resistant sublines*

IC_50_ measurements for the sensitive A375 cell line (0.33 μM) and the resistant A375PR1 (1.95 μM) and A375VR4 (2.47 μM) sublines.

**Supplementary Figure 2** *PTEN and PTENP1 expression levels in BRAFi resistant sublines relative to A375 cell line*

**(a)** Quantification of western blot (from Figure 1a) using the ImageJ software and corresponding full-length blots. **(b-d)** qRTPCR measuring the expression levels of (b) *PTEN,* (c) *PTENP1-AS* and (d) *PTENP1-S* in BRAFi resistant A375 sublines relative to A375 cells.

**Supplementary Figure 3.** *Expression levels of PTEN and PTENP1-AS isoforms in A375 cells and A375PR1 subline*

Full-length agarose gel blots measuring expression levels of *PTEN* and *PTENP1-AS* isoforms using semi-qRTPCR (related to figure 1e).

**Supplementary Figure 4** *Expression level of PTENP1-S*

qRTPCR analysis of the expression levels of *PTENP1-S* upon gapmer ASO-mediated knockdown of *PTENP1-AS*.

**Supplementary Figure 5** *Individual and simultaneous knockdown of EZH2 and DNMT3A*

**(a-b)** qRTPCR measuring the expression levels of (a) *EZH2* and (b) *DNMT3A* upon siRNA-mediated knockdown *EZH2* and *DNMT3A,* respectively. (**c-d**) qRTPCR measuring the expression levels of (c) *EZH2* and (d) *DNMT3A* in A375 and A375PR1 cells upon simultaneous knockdown *of EZH2* and *DNMT3A.*

**Supplementary Figure 6**

qRTPCR analysis of the expression levels of *PTENP1-S* upon dsiRNA-mediated knockdown of *C/EBPB*.

**Supplementary Figure 7** *Effect of the knockdown of PTENP1-AS on the colony formation of melanoma cell lines upon treatment with vemurafenib*

Colony formation of sensitive (A375) and resistant (A375PR1, A375VR4) melanoma cell lines evaluating the effect of knockdown of *PTENP1-AS* upon treatment with vemurafenib.

**SUPPLEMENTARY TABLE 1**

*Sequences of primers, siRNAs and antisense oligos*

| **qRTPCR** |  |
| --- | --- |
| B-actin F | AGGTCATCACCATTGGCAATGAG |
| B-actin R | CTTTGCGGATGTCCACGTCA |
|  |  |
| DNMT3a F | TTTGAGTTCTACCGCCTCCTGCAT |
| DNMT3a R | GTGCAGCTGACACTTCTTTGGCAT |
|  |  |
| EZH2 F | CAGTTTGTTGGCGGAAGCGTGTAA |
| EZH2 R | AGGATGTGCACAGGCTGTATCCTT |
|  |  |
| PTEN set I F (3'UTR) | AGA AAG CTT ACA GTT GGG CCC TGT |
| PTEN set I R (3'UTR) | GCC ACA GCA AAG AAT GGT GAT GCT |
|  |  |
| PTEN set II F (orf) | GGG ACG AAC TGG TGT AAT GAT ATG |
| PTEN set II R (orf) | CCA GAT GAT TCT TTA ACA GGT AGC TAT AA |
|  |  |
| PTENP1-AS F | CCTCACAGCGGCTCAACATTCAAA |
| PTENP1-AS R | AGGCTTCCAGGTTGGAAAGGAA |
|  |  |
| PTENP1-S F | AGTCACCTGTTAAGAAAATGAGAAGACAAA |
| PTENP1-S R | CTGTCCCTTATCAGATACATGACTTTCAA |
| C/EBPB set 2 F  C/EBPB set 2 R  **Detection of *PTENP1-AS*** | CGCGACAAGGCCAAGAT  GCTGCTCCACCTTCTTCTG |
| PTENP1-AS F0 | AAG CCC ACG GCT TCC ACC TT |
| PTENP1-AS F2 | AGACGAAGAAGAAGCGAGAAACGC |
| PTENP1-AS F3 | CCTCACAGCGGCTCAACATTCAAA |
| PTENP1-AS R0 | GCT GCA ATA ATC AAC AGA GTG TGG |
| PTENP1-AS R3 | AGGCTTCCAGGTTGGAAAGGAA |
|  |  |
| **ChIP** |  |
| PTEN pro F | TGATGTGGCGGGACTCTTTATGC |
| PTEN pro R | TCACAGCGGCTCAACTCTCAAACT |
|  |  |
| **siRNAs** |  |
| EZH2 #1 | Cat#; SI02665166 (Qiagen) |
| EZH2 #2 | Cat#; SI00063959 (Qiagen) |
| DNMT3a #1 | Cat#; SI02665271 (Qiagen) |
| DNMT3a_11 | Cat#; SI02665278 (Qiagen) |
|  |  |
| ***PTENP1-AS* siRNA** |  |
| Sense | GACGAAGAAGAAGCGAGAAAC TT |
| Antisense | pho-GUUUCUCGCUUCUUCUUCGUC TT |
|  |  |
| **DsiRNA** |  |
| C/EBPB | AGUUGAUGCAAUCGGUUUAAACATG |
|  | CAUGUUUAAACCGAUUGCAUCAACUUC |
|  |  |
| **GAPMER ASOs** |  |
| Gapmer Control | +C*+G*+A*A*T*A*G*T*T*A*G*T*A*+G*+C*+G |
| Gapmer *PTENP1-AS* | +C*+G*+T*+A*C*A*G*A*T*A*A*G*A*G*G*A*T*+T*+A |
